# Poly-L-lactic Acid (PLLA)-Chitosan-Collagen Electrospun Tube for Vascular Graft Application

**DOI:** 10.3390/jfb9020032

**Published:** 2018-04-30

**Authors:** Iffa A. Fiqrianti, Prihartini Widiyanti, Muhammad A. Manaf, Claudia Y. Savira, Nadia R. Cahyani, Fitria R. Bella

**Affiliations:** 1 Biomedical Engineering, Faculty of Science and Technology, Universitas Airlangga, Jawa Timur 60115, Indonesia; iffa.aulia-13@fst.unair.ac.id (I.A.F.); muhammad.abdul.manaf-13@fst.unair.ac.id (M.A.M.); claudia.yolanda-13@fst.unair.ac.id (C.Y.S.); nadia.rifqi.cahyani-14@fst.unair.ac.id (N.R.C.); fitria.renata.bella-2014@fst.unair.ac.id (F.R.B.); 2 Institute of Tropical Disease, Universitas Airlangga, Jawa Timur 60115, Indonesia

**Keywords:** poly L-lactic acid, collagen, chitosan, electrospinning, tube, vascular graft

## Abstract

Poly-L-Lactic acid (PLLA) blended with chitosan and collagen was used to fabricate a conduit for blood vessel engineering through an electrospinning process. Various concentrations of chitosan were used in the blend in order to study its effect on the morphology, chemical bond, tensile strength, burst pressure, hemocompatibility, and cell viability (cytotoxicity) of the tube.In vitro assessments indicated that addition of chitosan-collagen could improve cell viability and hemocompatibility. Best results were demonstrated by the conduit with 10% PLLA, 0.5% chitosan, and 1% collagen. Tensile strength reached 2.13 MPa and burst pressure reached 2593 mmHg, both values that are within the range value of native blood vessel. A hemolysis percentage of 1.04% and a cell viability of 86.2% were obtained, meeting the standards of high hemocompatibility and low cytotoxicity for vascular graft material. The results are promising for further development toward vascular graft application.

## 1. Introduction

Cardiovascular diseases (CVDs) are the leading cause of non-communicable disease death. In 2014, the WHO reported that CVDs killed 17.5 million people worldwide, 46% of total non-communicable deaths. The number of CVD sufferers has increased over the last ten years and will continue to grow. CVDs such as coronary heart disease, heart failure, and stroke are progressive outcomes of atherosclerosis, a complex pathological process of blood vessel wall hardening by lipo-protein deposits such as fat, cholesterol, cell waste products, calcium, and fibrin in the lumens or walls of large-sized (arterial) vessels [1].

Curative measures have not been successful due to limited success and experience intervening in the pathway mechanism of atherogenesis [1]. Thus, atherosclerosis is commonly treated with vascular graft bypass [2]. Material for vascular grafts must be biocompatible, non-thrombogenic, hemocompatible, and have suitable mechanical and physical characteristics [2]. Synthetic vascular grafts have gained popularity among medical practitioners for their ease of fabrication and handling. However, rejection of synthetic grafts due to thrombogenic re-occlusion is still a risk [3]. Thrombogenesis happens when endothelial tissue hyperplasia occurs as a result of mechanical characteristic mismatches between the graft and native vessel [3]. Most commercial synthetic grafts are based on non-degradable polymers (e.g., Dacron, ePTFE), which cannot be decomposed by the human body [4]. In the long run, the graft will maintain its original form, while the surrounding tissue continues to heal, increasing the chance of mechanical failure. On the other hand, degradable polymers (e.g., PLLA, PGA, PCL) allow the surrounding tissue to replace the graft as it proliferates over time, promoting graft-tissue integration and eventually completing revascularization [4].

Poly-L-Lactic Acid (PLLA) is a commonly used degradable synthetic polymer in tissue engineering. PLLA decomposes into lactic acid, which is processable through the Krebs cycle [5]. However, PLLA cannot be used alone in implant devices, because its hydrophobic nature would hinder interaction with host cells. To increase biocompatibility, natural polymers can be added. Chitosan is a natural polymer (polysaccharide) well known for its high biocompatibility and anti-bacterial properties. Chitosan can improve cell attachment to the surface of PLLA implants by lowering the hydrophobic properties of PLLA [6]. Collagen is another natural polymer found in extracellular matrix components. As the natural building block of extracellular matrices, collagen provides a suitable environment for cell attachment [7]. These three polymers have been used in various tissue engineering applications, such as skin tissue engineering, that include vascular conduit formation. Yin et al. fabricated vascular grafts using P(LLA-CL)-chitosan-collagen and found high suitability for the intended application [8].

Electrospinning is a method that can fabricate fine fibers in various structures. Fibrous structures have high surface-area-to-volume ratios, and can potentially mimic the extracellular matrix for cell attachment. PLLA, chitosan, and collagen were chosen to make tubes that possess mechanical strength as well as biocompatibility using the electrospinning process. The objective of this study was to examine the effect of chitosan concentration on the tube characteristics, including chemical characteristics (formation of chemical bond between PLLA, chitosan, collagen), fiber morphology, tensile strength, burst pressure, cytotoxicity, and hemocompatibility.

## 2. Results

### 2.1. Tube

**Figure 1:**
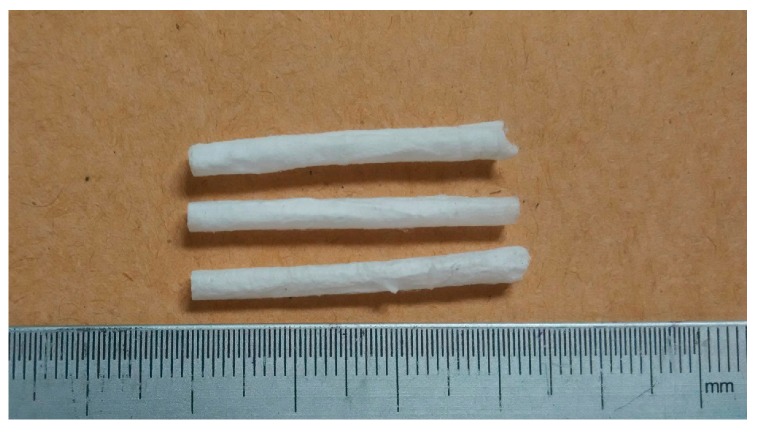
Tubes.

Tubes (Figure 1) in similar shapes to that of blood vessels were produced from electrospun polymer solution. There were three variations of tubes based on the composition of the solutions: sample A, from 10% PLLA; sample B from 0.5% chitosan, 1% collagen, and 10% PLLA; and sample C, from 0.6% chitosan, 1% collagen, and 10% PLLA.

### 2.2. FTIR Spectra of Tubes

**Figure 2:**
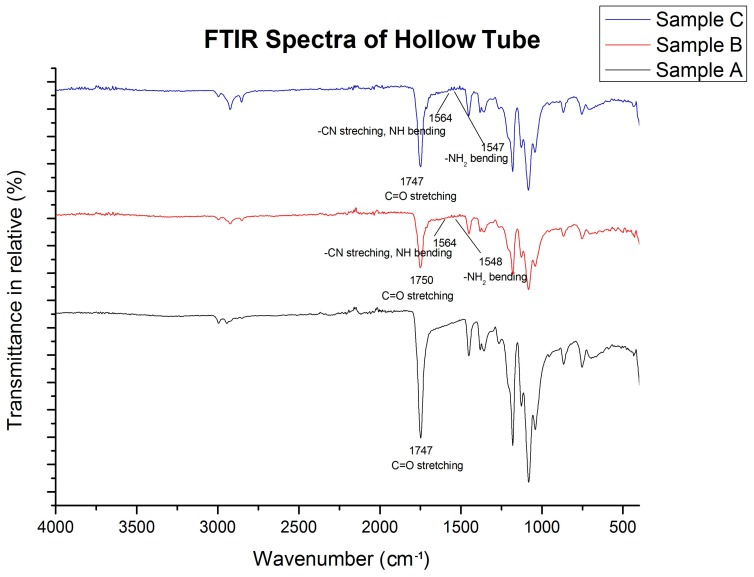
FTIR Spectra of Tube.

FTIR spectra (Figure 2) of sample B (0.5%:1%:10%, Chi:Col:PLLA) and C (0.6%:1%:10%, Chi:Col:PLLA) showed peaks corresponding to functional groups of PLLA (ester), Chitosan (Amine), and Collagen (Amide). The peak transmittance of the amine functional groups (–NH_2_ bending) from chitosan was present in the 1548 cm^−1^ band in sample B (0.5%:1%:10%, Chi:Col:PLLA) and 1547 cm^−1^ in sample C (0.6%:1%:10%, Chi:Col:PLLA). Amide II of collagen (C–N stretching, N–H bending) was present on the 1564 cm^−1^ band in both sample B and sample C (0.5%:1%:10%; 0.6%:1%:10%: Chi:Col:PLLA). All three samples had functional groups of PLLA (aliphatic C=O stretching) at 1750 cm^−1^ [9].

### 2.3. Morphology

**Figure 3:**
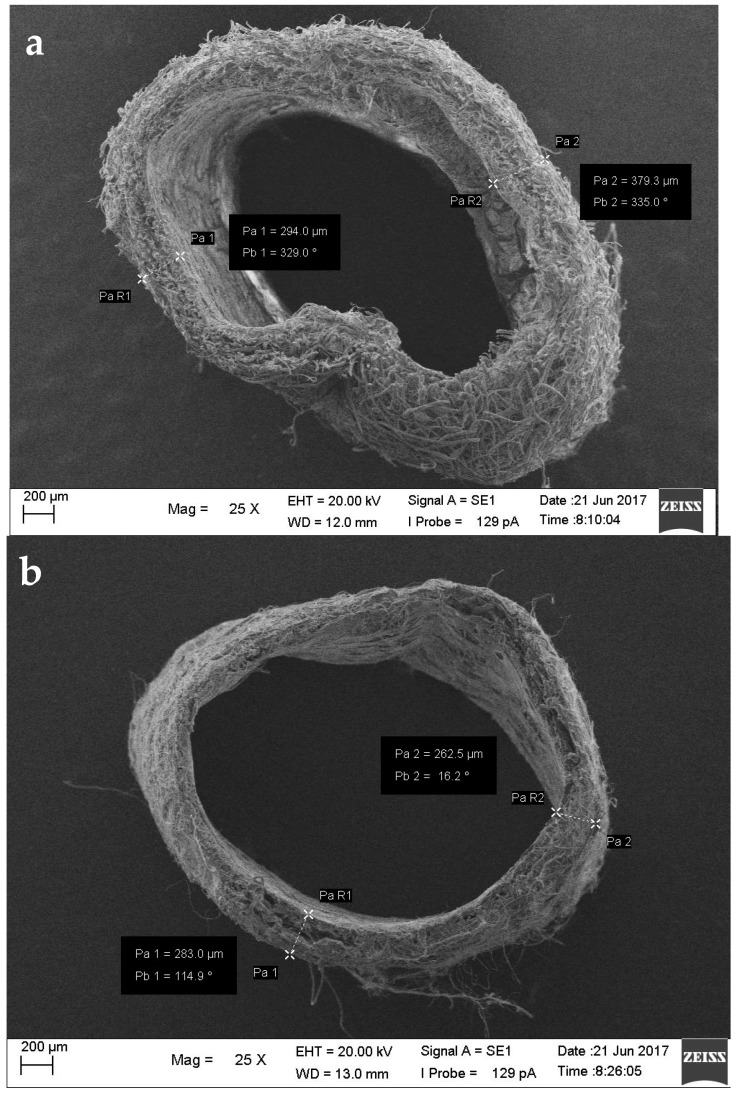

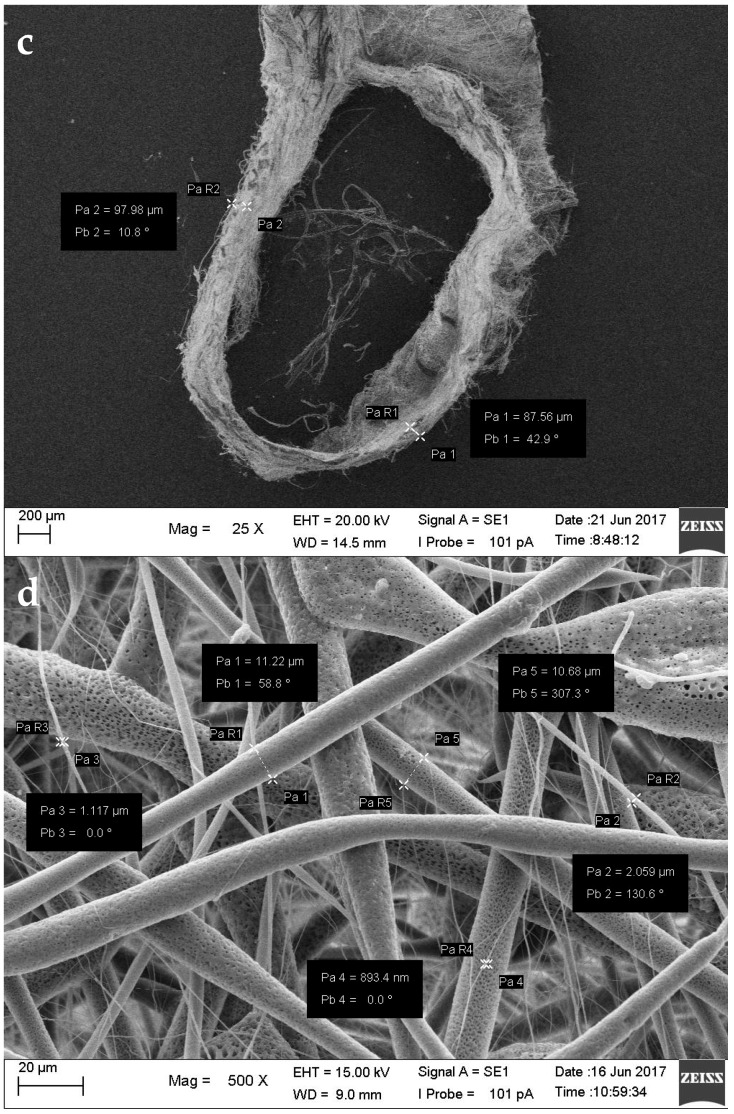

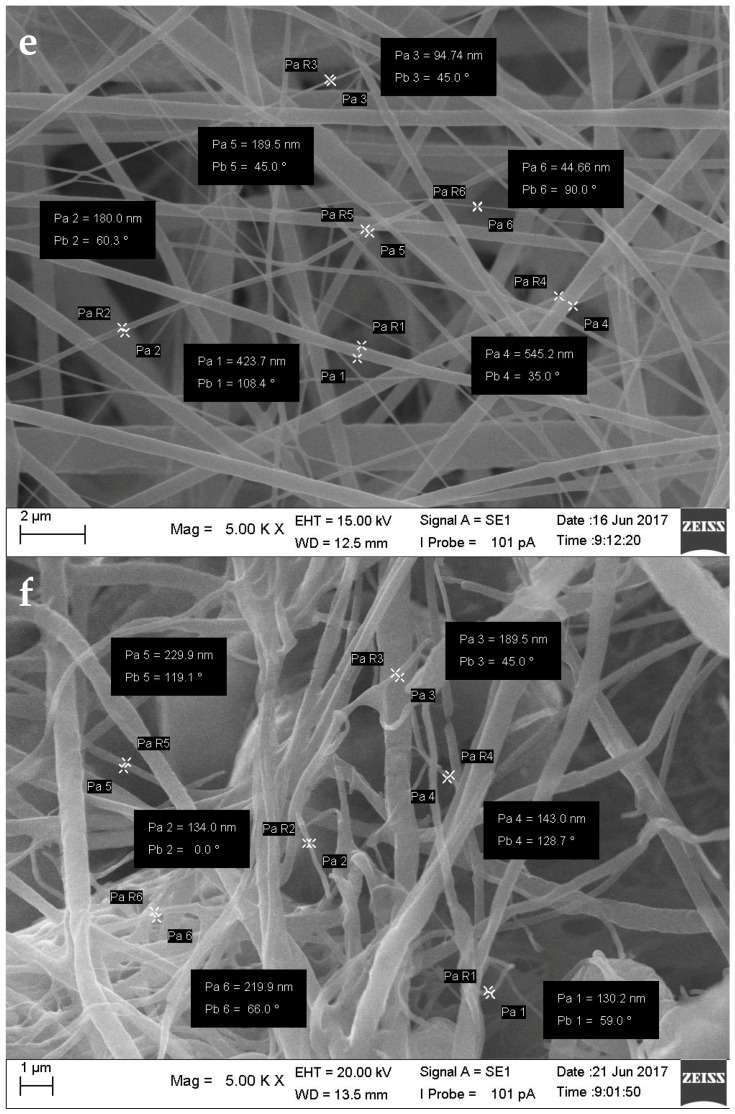
Cross-section (25× magnification) of (**a**) Sample A; (**b**) Sample B; (**c**) Sample C. Nanofiber diameters of (**d**) Sample A (500× magnification); (**e**) Sample B; (**f**) Sample C (5000× magnification), Pa is nanofiber diameter, Pb is the inclination of the measuring line.

Morphological assessment aimed to observe the diameter, thickness, fiber size, and fiber-to-fiber distance of the tube using scanning electron microscope (SEM) imaging (Figure 3). Mimicking the size of blood vessels in the tubes helps maintain the stability of blood flow, while the porous morphology facilitates cell attachment when reendothelialization occurs. The outer diameter of sample B (0.5%:1%:10%; Chi:Col:PLLA) was 2.93 ± 0.05 mm (Figure 3). The diameters of the tubes were in the range of heart coronary artery size at 2.97 ± 0.37–4.08 ± 0.44 mm [10]. The fiber-to-fiber distance was within the μm range (Table 1), where a pore size 20–30 μm is optimal for vascular prosthesis applications [11]. 

### 2.4. Mechanical Characteristics

**Figure 4:**
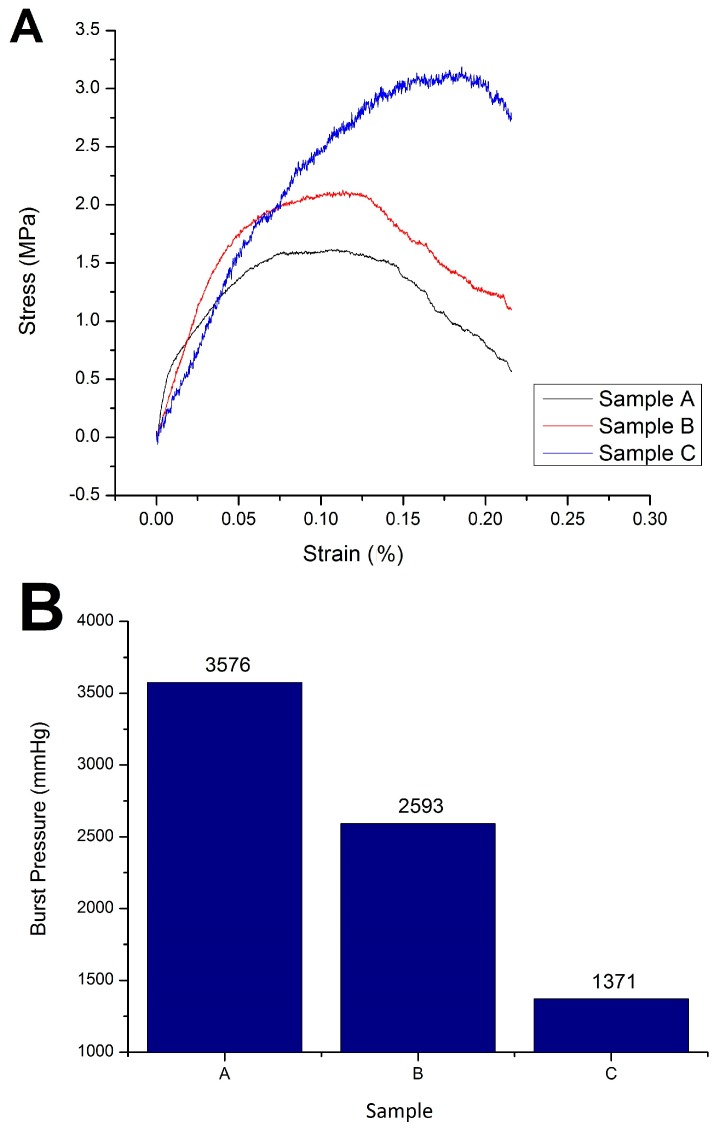
(**A**) Stress-strain curve of sample A, B, and C; (**B**) Burst pressure of sample A, B, and C.

A tensile test was used to determine the following mechanical properties of the tube: ultimate tensile strength and burst pressure (Table 2). Suitable mechanical properties are required by the vascular graft in order to maintain the lumen diameter under blood-flow pressure. Stress-strain curves (Figure 4) were used to determine ultimate tensile strength (UTS) and to calculate burst pressure. A UTS value of control sample A (0:0:10%; PLLA) was obtained at 1.62 MPa; sample B (0.5%:1%:10%; Chi:Col:PLLA) at 2.13 MPa; and sample at C (0.6%:1%:10%; Chi:Col:PLLA) at 3.19 MPa. These results correspond to the values reported by Karimi et al., in which the UTS of adult coronary arteries ranged from 1.46–3.20 MPa. The burst pressure value of the samples also decreased with increased chitosan concentration [12]. Burst pressure was calculated at 3576 mmHg for sample A (0:0:10%; PLLA), 2593 mmHg for sample B (0.5%:1%:10%; Chi:Col:PLLA), and 1371 mmHg for sample C (0.6%:1%:10%; Chi:Col:PLLA). These values are in accordance with Konig et al.’s study, which stated that the human arterial burst pressure is within the range of 1264–3196 mmHg [13].

### 2.5. In Vitro Cell Viability

**Figure 5:**
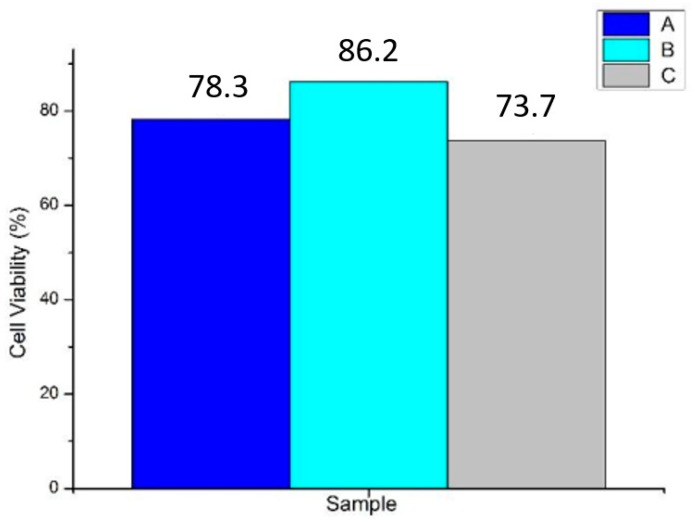
Cell viability in each sample A, B, and C.

A cell viability test was carried out to determine the cytotoxicity of the tubes, depending on the percentage of viable cells after incubation. The data is presented as an absorbance graph with respect to time, indicating the percentage of living cells over a given time span during incubation (Figure 5). The results after two hours showed cell viability in control sample A (0:0:10%, PLLA) of 78.3%; in sample B (0.5%:1%:10%; Chi:Col:PLLA) of 86.2%; and in sample C (0.6%:1%:10%; Chi:Col:PLLA) of 73.7%. Biocompatible materials have cell viability percentages above 50% [14]. Therefore, it can be concluded that all samples were non-toxic.

### 2.6. Hemolysis Assay

A hemolysis assay was conducted to determine the hemocompatibility of the tube. Hemocompatibility is the material’s ability to maintain the physiological condition of blood components during direct contact. The hemolysis assay reviews whether a tube can trigger a rupture of red blood cells (hemolysis). It was found that sample A (0:0:10%, PLLA) could trigger hemolysis; while sample B (0.5%:1%:10%; Chi:Col:PLLA) and sample C (0.6%:1%:10%; Chi:Col:PLLA) did not trigger hemolysis, with percentages of hemolysis of 14.63%, 1.04% and 3.14%, respectively (Table 3). Samples with chitosan and collagen in them corresponded to ASTM-issued criteria, which state that material with hemolysis percentages below 5% are declared safe for direct contact with blood (ASTM-F756-00).

## 3. Discussion

Current commercialized vascular grafts are mainly made from ePTFE, which cannot be degraded in the human body [4]. This research provides a reference of fabricating vascular grafts from biodegradable polymer poly-lactic acid (PLLA), chitosan, and collagen, which would be safe for application in the human body, since the residual product of degradation process can be processed by the system in the krebs cycle [5]. The graft can act as a scaffold for new vascular tissue growth on the surface of the vascular graft. Furthermore, the fibrous structure of the graft provides more spaces for new cell growth, compared to smooth-surfaced grafts [3]. 

Characterizations of the graft showed suitable results for vascular graft application, including fabricating process, morphology, mechanical strength, biocompatibility, and hemocompatibility. Ensuring the fabricating process was successful, the FTIR result showed distinct peaks of transmittance bands for each of the polymer contents, and these were ester for PLLA, amine for chitosan, and amide for collagen. The three peaks were found in each concentration variation of sample B (0.5%:1%:10%; chi:col:PLLA) and sample C (0.6%:1%:10%; chi:col:PLLA), however, the most prominent peaks were found in sample A, and belonged to the ester group, indicating that the only polymer in sample A was PLLA. There were no new peaks formed, indicating no bonds between PLLA, collagen, and chitosan. Sample B (0.5%:1%:10%; chi:col:PLLA) possessed the most suitable morphological features, with tubular diameter and wall thickness within the size range of the human coronary artery [9], and with pore size suitable for human vascular prostheses [10]. Sample A (control), and sample C were close to being suitable in terms of size. Mechanical strength (UTS and burst pressure) of all samples fall within the value of UTS and burst pressure of adult coronary artery according to Karimi et al. (2013) and Konig et al. (2009) respectively [12,13]. All samples showed good biocompatibility. In terms ofbiocompatibility, all samples had cell viability > 70%, with the highest being 86.2%, which was shown by sample B (0.5%:1%:10%; chi:col:PLLA). Sample A (0%:0%:10%; chi:col:PLLA) and C (0.6%:1%:10%; chi:col:PLLA) had cell viabilities of 78.3% and 73.3%, respectively. Only sample A (0%:0%:10%; chi:col:PLLA) triggered hemolytic events, with a hemolytic percentage of 14.63%. Sample B (0.5%:1%:10%; chi:col:PLLA) and sample C (0.6%:1%:10%; chi:col:PLLA) had hemolytic percentages of 1.04% and 3.14%, respectively, passing the criteria of hemolytic percentage allowed in vascular prostheses application issued in ASTM-F756-00.

Polymers breakdown through hydrolysis in the body, and their degradation is designed by improving their structure [15]. While this preliminary work could not fabricate an ideal vascular graft, it lays the foundation for future research in biodegradable grafts. This research could be expanded by implanting the vascular graft into a small animal model such as rabbit, moving onto a bigger mammalian model such as a canine or porcine model to obtain clinical data in the application of the vascular graft in the living organism. In the future, the vascular graft could be seeded by integrating cells from the patient’s own body into the vascular graft, ensuring a more reliable long-term interaction between the grafts with the surrounding tissue. Integrating the patient’s own cells would increase the ease of tissue growth on the graft.

## 4. Materials and Methods

### 4.1. Materials

Poly-L-lactic acid with molecular weight 100,000–125,000 Da was purchased from Polyscitech (Cat. No. AP06, Akina, IN, USA). Medium molecular weight, ~85% deacylated chitosan was purchased from Sigma Aldrich (Prod. No. 448877, Sigma Aldrich, St. Louis, MO, USA). Collagen type I was synthesized from red snapper fishbone. Reagent-grade acetic acid (Fulltime Chemical) and chloroform (SAP Chemicals) were used for solvents.WST-1 assay reagent, phosphate-buffered saline (PBS), fetal bovine serum (FBS), dimethyl sulfoxide (DMSO), human blood sample, and lymphocyte cell T (MOLT-4) were also used.

### 4.2. Electrospinning

Firstly, electospinning dope was prepared. Each polymer was dissolved separately in room temperature for 6 h, stirred in 500 rpm. 10% PLLA was dissolved in chloroform, collagen and chitosan in 0.5 M acetic acid. The collagen and chitosan solutions were made first by testing their solubility in 0.5 M acetic acid. The collagen solution reached homogeneity at 1% concentration, whereas the chitosan solution reached homogeneity at 0.6%. Hence, the collagen concentration chosen was 1%, and the chitosan concentrations chosen were 0.6% and 0.5%. The three solutions (i.e., chitosan, collagen, and PLLA) were stirred at room temperature in a fixed volume ratio of 5:5:90 (chi:col:PLLA) for 12 h at 700 rpm. Three different solutions were made, the control solution was 10% PLLA alone (sample A), the first variation was chi:col:PLLA = 0.5%:1%:10% (sample B), and the second variation was chi:col:PLLA = 0.6%:1%:10% (sample C) (Table 4).

The electrospinning dope was placed in a glass syringe using a 23 G blunt-tip needle above the collector, a rotating stainless-steel rod (diameter = 2 mm, rotation = 3000 rpm). Electrospinning was conducted in 15 kV electric field, 0.5 mL/h flow rate for 4 h, and 12 cm tip-to-collector distance using Nabond Nano E-Spinning Unit. Flat (membrane) samples were also made for the hemolysis assay by changing the stainless-steel rod collector into aluminum foil sheet.

### 4.3. Evaluation

#### 4.3.1. Fourier Transform InfraRed (FTIR)

Each tube sample variation was cut into tubes with 2 mm length. Samples were ground and prepared using the KBr disc method, a Bruker α FTIR instrument (Bruker, Billerica, MA, USA) was used to obtain the infrared spectra of the tubes. The infrared used was 4000–400 cm^−1^.

#### 4.3.2. Mechanical Testing

Shimadzu AGS-X (Shimadzu Corp., Kyoto, Japan) was used for tensile testing of the electrospun tube, conducted at a temperature of 25.9 °C, and 61% room humidity. Samples were prepared using a template described in Huang et al. [16]. Each sample variation was cut into tubes 3 cm in length, and then dipped into phosphate buffer saline to illustrate the state of graft in the body. The tube was clamped at both ends, with a piece of steel rod inside the tube along the clamped parts to maintain its tubular form. Gauge length was 1cm, the load cell was 5 kN, then pulled at 5mm/min speed. The force-displacement curve was plotted in real time using Trapezium X, and later processed into the stress-strain curve in Origin 8.5. The burst pressure was indirectly determined using the following formula [17]:

$$Burst\_pressure=\frac{2\times UTS\times Thickness}{Internal\_Diameter}$$

#### 4.3.3. Morphology

Zeiss Scanning Electron Microscopy (Zeiss, Oberkochen, Germany) was used to observe fiber morphology, including tube diameter, fiber diameter, and fibers-to-fibers distance. The samples were prepared by cutting the tube, and then sputtered with palladium-gold. The samples were observed cross-sectionally, under 25 times, 500 times, and 5000 times magnification.

#### 4.3.4. Cell Viability Test (Cytotoxicity)

The cell viability test was performed with WST-1 assay method using a lymphocyte T cell. Samples were cut into 5mm-length tubes and dissolved in DMSO, and then mixed with the cell in well plates containing a medium of RPMI, 10% FBS, and 5% natrium bicarbonate protocol. Samples were treated with WST-1 reagents and then incubated. The color change was assessed using spectrophotometry with a wavelength of 450 nm in the time span of 0 m, 30 m, 1 h, and 2 h. The percentage of living cells was calculated using the following formula [18]:

$$\%Viable\_cell=\frac{Sample\_absorbance-Media\_absorbance}{Cell\_absorbance-Media\_absorbance}\times 100\%$$

#### 4.3.5. Hemolysis Assay

The hemolysis assay was performed by comparing light absorbance (wavelength of 490 nm) between negative control solution (100 μL of blood dissolved in 10 mL PBS) and positive control solution (100 μL of blood dissolved in 10 mL distilled water). Samples (3 × 3 mm) were mixed in negative control solution and incubated for two hours at 37 °C; then, light absorbance was assessed by microplate reader. Hemolysis percentage was obtained by the following formula [19]:

$$\%Hemolytic=\frac{Sample\_absorbance-Negative\_control\_absorbance}{Positive\_control\_absorbance}\times 100\%$$

## 5. Conclusions

Preparation of the tube was successful, from amine and amide function groups detected in sample B (chitosan 0.5%; Collagen 1%; and PLLA 10%) and sample C (0.6% chitosan; collagen 1%; and PLLA 10%) on wave number 1548 cm^−1^, 1547 cm^−1^ (amine), and 1564 cm^−1^ (amide); in addition, ester C=O stretching was also detected at 1747 cm^−1^, and 1750 cm^−1^respectively. Control sample and variation samples with increasing chitosan concentration tensile strength of 1.62 MPa, 2.13 MPa, and 3.19 MPa; and burst pressure of 3576 mmHg, 2593 mmHg, and 1371 mmHg. All variations of vascular graft tubes had μm-sized fiber-to-fiber distance suitable for vascular prosthesis applications. The tube was non-toxic, with viable cell percentages on sample A (control) at 78.3%; sample B at 86.2%; and sample C at 73.7%; thus, it was deemed safe for direct contact with blood with hemolysis for sample B at 1.04%, and sample C at 3.14%. Of all variations of vascular graft tube, the best characteristics were possessed by sample B.

## Figures and Tables

**Table 1:** The results of morphological study of the vascular grafts.

Sample	Outer Diameter (mm) and Thickness (mm)	Fiber Diameter (nm)	Fiber-to-Fiber Distance (µm)
A (PLLA)	3.04 ± 0.02 0.39 ± 0.03	135.8–205.9	3.796–31.27
B (PLLA-Collagen-Chitosan 0.5%)	2.93 ± 0.05 0.22 ± 0.03	89.33–246.7	5.141–30.144
C PLLA-Collagen-Chitosan 0.6%)	2.87 ± 0.06 0.08 ± 0.08	130.2–229.9	4.362–30.872

**Table 2:** Tensile strength and burst pressure of Sample A, B, and C.

Sample	Tensile Strength (N/mm^2^)	Burst Pressure (mmHg)
A (Control)	1.62	3576
B (PLLA-Collagen-Chitosan 0.5%)	2.13	2593
C (PLLA-Collagen-Chitosan 0.6%)	3.19	1371

**Table 3:** Absorbance and hemolysis status in sample A, B, and C.

Sample	Average Absorbance (Hm)	Hemolysis Percentage (%)	Hemolytic Grade
A (PLLA, Control)	0.052	14.63	Hemolytic
B (PLLA-Collagen-Chitosan 0.5%)	0.039	1.04	Non Hemolytic
C (PLLA-Collagen-Chitosan 0.6%)	0.041	3.14	Slightly Hemolytic

**Table 4:** Concentration of each polymer in the samples.

Sample	Concentration (*w*/*v* %)		
	PLLA	Collagen	Chitosan
A (PLLA, Control)	10	0	0
B (PLLA-Collagen-Chitosan 0.5%)	10	1	0.5
C (PLLA-Collagen-Chitosan 0.6%)	10	1	0.6
